# The Development of a Chatbot Technology to Disseminate Post–COVID-19 Information: Descriptive Implementation Study

**DOI:** 10.2196/43113

**Published:** 2023-06-05

**Authors:** Mayssam Nehme, Franck Schneider, Anne Perrin, Wing Sum Yu, Simon Schmitt, Guillemette Violot, Aurelie Ducrot, Frederique Tissandier, Klara Posfay-Barbe, Idris Guessous

**Affiliations:** 1 Division of Primary Care Medicine Geneva University Hospitals Geneva Switzerland; 2 Department of Communication Geneva University Hospitals Geneva Switzerland; 3 Division of General Pediatrics Geneva University Hospitals Geneva Switzerland; 4 Faculty of Medicine University of Geneva Geneva Switzerland

**Keywords:** COVID-19, post–COVID-19, long COVID, PASC, postacute sequelae of SARS-CoV-2, chatbot, medical technology, online platform, information, communication, dissemination, disease management, conversational agent, digital surveillance, pediatric, children, caregiver

## Abstract

**Background:**

Post–COVID-19, or long COVID, has now affected millions of individuals, resulting in fatigue, neurocognitive symptoms, and an impact on daily life. The uncertainty of knowledge around this condition, including its overall prevalence, pathophysiology, and management, along with the growing numbers of affected individuals, has created an essential need for information and disease management. This has become even more critical in a time of abundant online misinformation and potential misleading of patients and health care professionals.

**Objective:**

The RAFAEL platform is an ecosystem created to address the information about and management of post–COVID-19, integrating online information, webinars, and chatbot technology to answer a large number of individuals in a time- and resource-limited setting. This paper describes the development and deployment of the RAFAEL platform and chatbot in addressing post–COVID-19 in children and adults.

**Methods:**

The RAFAEL study took place in Geneva, Switzerland. The RAFAEL platform and chatbot were made available online, and all users were considered participants of this study. The development phase started in December 2020 and included developing the concept, the backend, and the frontend, as well as beta testing. The specific strategy behind the RAFAEL chatbot balanced an accessible interactive approach with medical safety, aiming to relay correct and verified information for the management of post–COVID-19. Development was followed by deployment with the establishment of partnerships and communication strategies in the French-speaking world. The use of the chatbot and the answers provided were continuously monitored by community moderators and health care professionals, creating a safe fallback for users.

**Results:**

To date, the RAFAEL chatbot has had 30,488 interactions, with an 79.6% (6417/8061) matching rate and a 73.2% (n=1795) positive feedback rate out of the 2451 users who provided feedback. Overall, 5807 unique users interacted with the chatbot, with 5.1 interactions per user, on average, and 8061 stories triggered. The use of the RAFAEL chatbot and platform was additionally driven by the monthly thematic webinars as well as communication campaigns, with an average of 250 participants at each webinar. User queries included questions about post–COVID-19 symptoms (n=5612, 69.2%), of which fatigue was the most predominant query (n=1255, 22.4%) in symptoms-related stories. Additional queries included questions about consultations (n=598, 7.4%), treatment (n=527, 6.5%), and general information (n=510, 6.3%).

**Conclusions:**

The RAFAEL chatbot is, to the best of our knowledge, the first chatbot developed to address post–COVID-19 in children and adults. Its innovation lies in the use of a scalable tool to disseminate verified information in a time- and resource-limited environment. Additionally, the use of machine learning could help professionals gain knowledge about a new condition, while concomitantly addressing patients’ concerns. Lessons learned from the RAFAEL chatbot will further encourage a participative approach to learning and could potentially be applied to other chronic conditions.

## Introduction

### Background

Post–COVID-19 [1], or long COVID, describes the persistence of symptoms following a SARS-CoV-2 infection. Post–COVID-19 is prevalent in 10%-30% of outpatient adult individuals [2,3] and can potentially transform into a chronic condition, with patients suffering from symptoms over months to years. Additionally, new studies have shown that children can suffer from post–COVID-19 as well [4-8], even though the prevalence is overall lower in the pediatric population and can vary between 2% and 9%. In Geneva, Switzerland, the CoviCare follow-up program is a longitudinal outpatient cohort established in March 2020 [9], following the long-term evolution of symptoms after SARS-CoV-2 infection and their impact on daily life. Results initially showed a third of infected individuals still had symptoms at 6 weeks [9], then at 7-9 months [2], and at 12 months [10]. Although the prevalence of symptoms decreased with time, 33% of patients still had symptoms at 12 months: 16% reported fatigue, 10% had a loss of taste or smell, 9% had persistent dyspnea, 10% had headaches, 9% had sleep disorders, 7% had difficulty concentrating, and 7% had myalgia. In young children and adolescents, studies have shown the persistence of symptoms beyond 3 months and potentially with long-term effects, especially in adolescents [6,8]. To date, there is little information about the long-term evolution and treatment options for post–COVID-19, and symptoms may persist for years in a percentage of patients, with a potential impact on daily life.

The uncertainty of knowledge and the fast-growing numbers of patients prompted affected individuals and health care professionals alike to seek information online, on social network groups, and by contacting medical and public health representatives. Post–COVID-19 groups began to emerge worldwide, with information varying in its accuracy at times. In Geneva, to better assist health care professionals in their learning approach and management of patients with SARS-CoV-2 infection, the CoviCare program created algorithms and guidelines for the management of acute and long-term complications of COVID-19 [11-13] and shared them online with all health care professionals on local and international levels. In November 2020, a post–COVID-19 interdisciplinary adult consultation was established in the Division of Primary Care Medicine at the Geneva University Hospitals Geneva, Switzerland [14], followed by an interdisciplinary pediatric consultation in the Division of General Pediatrics in May 2021 [15]. The increasing demand for consultations and answers in a time of limited resources made it challenging to manage all requests. Additionally, several remote patients started contacting the post–COVID-19 clinic, not able to find answers locally. A growing need for the awareness and public recognition of symptoms and their impact became evident as patients struggled to get care and to explain their symptoms to their families, employers, schoolteachers, and surroundings in general.

An innovative tool to answer these challenges was needed in a time of limited resources, and that is when the post–COVID-19 group at the Geneva University Hospitals turned to chatbot technology as an approach to respond to these needs. Chatbots are an easy-to-use interactive tool that uses natural language to provide access to information 24-7. Their scalability and wide accessibility make them perfect tools to disseminate information and substitute in-person contact, while providing an interactive exchange [16]. The use of chatbots to provide medical information [17,18] had begun before the COVID-19 pandemic; however, their use was limited, and evidence supporting this approach in clinical settings still needed to be established. Some of the initial concerns were over their safety and efficacy in handling medical information [19,20]. In health care, chatbots can be informational or interventional. They can be used to provide medical information [17], schedule appointments [21], collect data, handle insurance inquiries, provide mental health assistance [22], and follow chronic diseases and interventions [23]. Their use in the management of the COVID-19 pandemic from a public health perspective has demonstrated their widespread utility from surveillance to information dissemination and coordination [16]. In multilingual countries, such as Switzerland, experiences with chatbot technology in local languages on multiple platforms (eg, Facebook, WhatsApp) has proven critical for people to access reliable information in their own language [24]. Chatbots also have the capability of integrating user information to improve their learning. This feature could prove to be especially favorable in contexts such as post–COVID-19, where professionals and patients have been learning at the same time about long-term symptoms, their evolution, and their impact on daily life [25].

RAFAEL [26], an online information and exchange platform, was deployed by the Geneva University Hospitals [26] to address specifically the postacute sequelae of SARS-CoV-2 (PASC) or post–COVID-19 in children and adults. To date, RAFAEL [26] is an ecosystem including online information grouped by symptoms and themes, chatbot technology, and regularly scheduled webinars. RAFAEL’s main goal is to answer the increasing demand for information and communication on post–COVID-19 in a time of limited knowledge and limited resources for both children and adults.

### Objective

Post–COVID-19 is a disease with a considerable knowledge gap to date, affecting a large proportion of individuals and impacting their health as well as social, professional, and family lives. RAFAEL [26], an online interactive platform with a chatbot feature, was aimed to develop, disseminate, and promote reliable information about post–COVID-19 in children and adults. The objective of this study is to describe and evaluate the implementation of a chatbot and to assess the accuracy of and user satisfaction with the chatbot regarding dissemination of information about post–COVID-19.

## Methods

### Ethical Considerations

No ethical committee approval was required at this stage as this study evaluated the development and implementation of a chatbot tool and all collected information was part of the quality assurance data when using a chatbot technology. This is in line with the local Commission for ethical research in Geneva, Switzerland.

### Study Setting and Participation

The RAFAEL study was conducted in Geneva, Switzerland, led by the Division of Primary Care Medicine, the Division of General Pediatrics, and the Department of Communication at the Geneva University Hospitals. The RAFAEL platform is in collaboration with several hospitals, academic institutions, legal experts, patients, and patient-led organizations in Switzerland and worldwide (to date, more specifically in the francophone world).

All participants are users of the RAFAEL chatbot (Table 1). User profiles are divided into information about adults or about children. Users who select information about adults can switch to information about children, and vice versa.

**Table 1:** Users of the RAFAEL chatbot.

User profile	Type of information
Individuals seeking information about adults	I have been diagnosed with post–COVID-19. I think I have post–COVID-19. I am a health care professional. Other.
Individuals seeking information about children	My child has been diagnosed with post–COVID-19. I think my child has post–COVID-19. I am a health care professional. Other.

### Chatbot Development Strategy

The development phase started in December 2020 after identifying the population-based need for seeking reliable information about post–COVID-19. At this stage, there was a sharp increase in post–COVID-19 consultations at the Division of Primary Care Medicine [14] and the staff was unable to address all individual needs. There were also early signals of potential post–COVID-19 in children [15]. Following the knowledge and information that the CoviCare cohort [10-12] provided about post–COVID-19, the Geneva University Hospitals and, more specifically, the RAFAEL team were looking for ways to actively engage and interact with patients and citizens to better understand post–COVID-19. Community and patient engagement has been a cornerstone of the conception, development, and deployment of the RAFAEL platform and chatbot. The RAFAEL team included patients with post–COVID-19 since the early stages, with scheduled meetings and feedback sessions (5 meetings and feedback sessions) to review questions and answers, as well as the content of the platform. Patients with post–COVID-19 were also invited to participate as speakers in all the webinars. The Geneva University Hospitals also has a group of information for patients and their families comprising 10 individuals (n=2, 20%, partner patients; n=2, 20%, nurses; n=2, 20%, physicians; and n=4, 40%, individuals from the communication department) whose role is to review all texts and content addressed to patients and the general population. This group has contributed to and revised the content of RAFAEL and the chatbot algorithms. Additionally, around 20 patients and parents of patients have participated in the beta testing, evaluating the chatbot’s use, ergonomic setup, and answers before deployment. Patients who are part of the Long Covid Association in Switzerland are also active partners of the RAFAEL platform and have contributed to and revised the content. The input of patients and the community has been essential in providing a community-friendly, useful, and appropriate platform that is easy to use and provides the required information. The setup of the RAFAEL platform and chatbot technology are described in Textbox 1.

The timeline and development strategy are described in Table 2.

The specific strategy behind the RAFAEL chatbot was specific in its concept, balancing ease of use and interaction with medical safety. Chatbots are computer programs simulating human conversations through voice or text interactions. Their intent is to be as close to a human interaction as possible and to let users believe they are having a conversation with a human being. By doing so, chatbots successfully fulfill the Turing test requirements [27]. The Turing test developed in 1950 by Alan Turing, a mathematician and computer scientist, is based on an “imitation game” and involves a machine and 2 participants (an evaluator and a responder). If a machine holds a conversation with a human being without being detected as such, the test is successful. The Turing test has become a source of inspiration for all engineers aiming to build machines with humanlike conversational capacities.

Our specific objectives were slightly different. To limit any confusion on the part of the users, we believed it was important that users be made aware that the chatbot was not a human being, especially when dealing with medical information. Our aim was for the chatbot to provide accurate answers and maximize user satisfaction by increasing transparency and making users completely aware of the use and usefulness of the tool at hand. The RAFAEL chatbot was depersonalized without displaying a name or an avatar. The chatbot did not include small talk, such as “How are you today?” or “Glad to talk to you today.” Moreover, with our approach focused on medical safety, the chatbot provided answers to the most frequently asked questions. Complex or more specific questions are redirected to a contact form, where a community moderator or health care professional answers within 24-48 hours.

To reduce errors, we used a selection of open conversations in natural language and scripted conversations. Through open conversations, users could freely ask their questions, which were reformulated by the chatbot to avoid misunderstandings. The users then received answers with a link that redirected them to the information about RAFAEL’s website. With scripted conversations, users could quickly and easily find information by selecting options.

**Setup, concept, and indicators of the RAFAEL platform and chatbot.**

**Developing the concept of the RAFAEL platform**

- Meetings and brainstorming with stakeholders, patients, experts in communication, physicians managing post–COVID-19 in children and adults, IT experts, and funders

**Project proposals**

- Static web pages with information
- Add-on chatbot technology
- Add-on webinars and monthly online meetings open to the general population

**Assessment**

- Needs
- Feasibility
- Time
- Resources
- Utility
- Framework
- Indicators
- Deployment/communication
- Maintenance
- Growth

**Indicators**

- Usability
- Accuracy
- Appropriateness of response
- Satisfaction
- Volume
- Themes
- Attractiveness/new users

**Table 2:** Timeline and development strategy.

Development strategy		December 2020-February 2021		March-June 2021		July-September 2021		October-November 2021		December 2021-February 2022
**Concept**										
	Use and need in post–COVID-19	Applicable	N/A^a^		N/A		N/A		N/A	
	Strategy: user safety vis-à-vis medical information	Applicable	N/A		N/A		N/A		N/A	
**Backend development**										
	Conversational guide	N/A	Applicable		N/A		N/A		N/A	
	Scenarios	N/A	Applicable		N/A		N/A		N/A	
	Algorithms	N/A	Applicable		N/A		N/A		N/A	
**Frontend development**										
	Design	N/A	N/A		Applicable		N/A		N/A	
	Integration	N/A	N/A		Applicable		N/A		N/A	
	Editing	N/A	N/A		Applicable		N/A		N/A	
**Beta testing**										
	Testing within the RAFAEL team and communication department	N/A	N/A		N/A		Applicable		N/A	
	Focus groups with adults	N/A	N/A		N/A		Applicable		N/A	
	Focus groups with teenagers	N/A	N/A		N/A		N/A		Applicable	

^a^N/A: not applicable.

### Chatbot Technology

The RAFAEL chatbot used natural language processing (NLP) and, more specifically, natural language understanding (NLU). This machine learning technique extracted context and meaning from user input (user query). The user input could be structured or unstructured. The chatbot identified an intent that mapped the conversion between user input and the action that the chatbot should then take to answer. The intent was identified in predefined formulations with entities (words, a concept or subject that could be used in a sentence). The first step was to give entities specific weights, with stronger weights for entities that could help define the sentence or intent of the person (eg, fatigue in post–COVID-19, malaise, feeling tired). As fatigue and postexertional malaise are key features of post–COVID-19, these words would be given a higher weight, considering the higher probability that users would be looking for information about these symptoms. The next step was to define synonyms as well as entities that were required or not required to match the intent. The intent was then given a priority level (minimum, low, medium, high, or maximum) and an intent-matching method. The intent-matching method could be (1) “Contains” (used if the objective was to match the intent only if all of the words or 1 of the formulations were contained in the user query), (2) “NLP engine” (used to let the engine match the user query and the intent), and (3) “Exact match” (matched the intent only when the user query was exactly the same as 1 of the formulations). The intents were then used in stories linking different steps to create an interactive response with the user. Stories were scenarios of answers that were triggered based on specific intents. This approach ensured that a user request did not match with 2 different stories.

**Figure 1:**
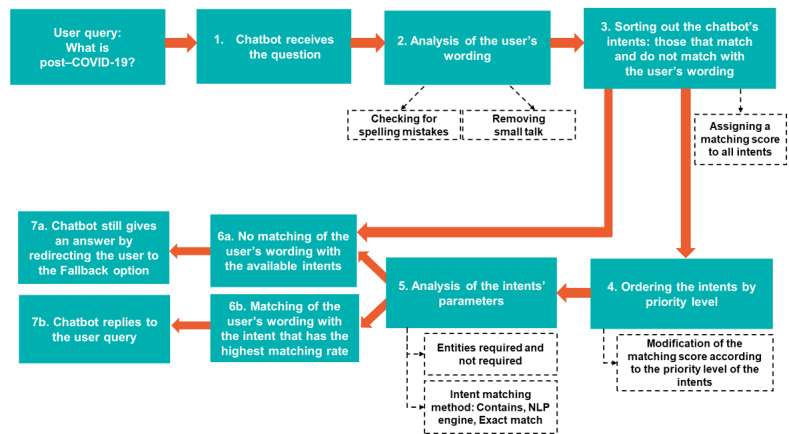
Chatbot algorithm and pathway from the initial user query to the final matching and answer. NLP: natural language processing.

The RAFAEL chatbot started off the conversation with a welcome scenario. Users were then prompted to use buttons to determine whether they were looking for information about children or adults. Users were also asked to identify whether they had post–COVID-19, thought they had post–COVID-19, were health care professionals, or belonged to the “other” category. Users entered their questions or concerns in open-ended format, and the chatbot analyzed the information to match an intent using the steps mentioned earlier. The chatbot used conditions determining the links between steps. Finally, is the RAFAEL chatbot could not process a question, it displayed a Fallback option, where users could click specific buttons, depending on the information they were requesting: (1) General information about post–COVID-19, (2) Diagnosis of post–COVID-19, (3) Listing of post–COVID-19 consultations, (4) Symptoms, (5) Information about COVID-19 in general, and (6) Contact form. If selected, the contact form then asked for the user’s name, email, and question and was sent via secure messaging to the community moderators or health care professionals of the RAFAEL team. A written answer was provided within 24-48 hours. The matching rate was considered based on the chatbot correctly identifying the keywords, entities, and intents and providing the appropriate answer or redirecting the user to the relative information page on the RAFAEL platform. With the lack of full knowledge around post–COVID-19, weekly monitoring of the questions and answers was established to ensure the appropriateness and accuracy of the answers provided. The weekly monitoring was performed by the RAFAEL moderating team comprising 1 digital communication expert, 1 physician with knowledge and experience in post–COVID-19 on the clinical and research levels, and 1 medical student. All questions and answers were reviewed, and each answer was evaluated for accuracy, completeness, and potential alternative answers. The satisfaction level was considered after each interaction, as users were asked whether their exchange was satisfactory and corresponded to their expectations. Each conversation ended with the following question to which the user could answer yes or no: “Did I answer your request correctly?” This answer helped the team evaluate the user’s satisfaction level in relation to the chatbot’s answer. If the user replied no to the question, they were encouraged to use the contact form to get in touch with the chatbot’s moderators. Figure 1 summarizes the algorithm and pathway used by the RAFAEL chatbot to understand and process the information and provide a final answer.

### Legal Aspects

All steps were anonymous, and no identifying information was collected except when using the contact form. The information in the contact form is stored on secure servers in Europe for 3 years. The chatbot technology and, specifically, the contact form respect the General Data Protection Regulation (GDPR) [28]. Prior to deployment, a thorough evaluation was conducted by legal experts with expertise in children’s rights. This was to ensure that the use of the platform was compatible with the regulations relative to children’s rights and that the guidelines governing the ethical use of technology were applied.

### Deployment, Partnerships, and Communication Strategy

Following the development phase, deployment started in November 2021. A deployment strategy was set up (Table 3) and was supported by partnerships and communication campaigns [29].

**Table 3:** Deployment strategy.

Strategy	November-December 2021	January-February 2022	March-June 2022	July-December 2022	January-February 2023	March-June 2023
Online deployment	Applicable	N/A^a^	N/A	N/A	N/A	N/A
Communication	Applicable	Applicable	N/A	N/A	N/A	N/A
Partnerships	N/A	Applicable	Applicable	Applicable	N/A	N/A
Continuous monitoring	Applicable	Applicable	Applicable	Applicable	Applicable	Applicable
Monthly webinars	N/A	Applicable	Applicable	N/A	Applicable	N/A

^a^N/A: not applicable.

### Partnerships and Community Engagement

Several institutional partnerships have been established to promote the use of the RAFAEL platform in different regions worldwide and to respond to specific population needs. To date, 15 institutions, including Swiss hospitals in the regions of Vaud, Valais, and Jura; large health care networks; the Swiss Society of General Internal Medicine; the Association of Pediatricians; the United Nations High Commissioner for Refugees (UNHCR); the International Committee of the Red Cross (ICRC); the Long COVID Association in Switzerland; and the University of Sherbrooke (Canada), are active partners of the RAFAEL platform. The partners have deployed the platform as an information tool on post–COVID-19 in their respective areas (geographic but also specific to staff in large organizations, members of scientific societies, etc) and attended regular meetings to share knowledge and information about post–COVID-19 and the RAFAEL technology. Increasing user numbers and reaching new audiences was critical for the RAFAEL chatbot as this diversified and multiplied the database provided to the chatbot via user interaction. Community engagement and working in partnerships with patients and patient-led organizations facilitated the development of a tool that was appropriate and easy to use for patients and the general population. In a disease where integrative medicine approaches have been used to date [30], and patients or health care professionals might have questions about a large spectrum of symptoms and management options, community engagement helped develop specific questions on the use of these therapies and whether they should be integrated in the clinical care pathway. The partnerships with the UNHCR and the ICRC were especially useful in addressing potential concerns on post–COVID-19 in French-speaking countries in Africa, the Caribbean, and South America. As access to care, specialists, and the clinical care pathway of patients might differ in these settings, a good knowledge of the field and partnerships with organizations that have a medical and social presence in these communities was important. The RAFAEL team tried to reach out to hospitals and health care centers in the field directly; however, these did not seem to have post–COVID-19 clinics or a specific pathway for patients. Ideally, the information provided about the RAFAEL platform can help them better identify and manage patients with similar symptoms.

### Communication

#### Communication Strategy and Goals

The first goal of the communication campaign was to inform the public and interested individuals and institutions of the existence and deployment of RAFAEL and to increase awareness around the platform and the chatbot. As the use of chatbot technology in clinical settings is still not easily adopted, the goal of increasing awareness about the advantages of using this technology was essential.

The communication strategy also aimed at developing ways to recruit and retain users. The self-learning aspect of the chatbot on post–COVID-19 was an essential part of the strategy, and it was therefore important to continually drive traffic to the website. The RAFAEL webinars were also an essential tool to help the online community discover and use the RAFAEL chatbot and platform in general.

In total, 5 monthly webinars were scheduled between February and June 2022 and 2 more in October and December 2022, with an average of 250 participants at each webinar. The webinars had interventions by patients and specialists from different fields, including medical professionals, physical therapists, occupational therapists, school representatives and representatives of employers, and the disability insurance network.

#### Communication Methods

We created visual designs associated with the RAFAEL platform and post–COVID-19. Knowing that post–COVID-19 is associated with a young age and the female sex, we opted for a young woman with fatigue as the main character in the RAFAEL communication tools. We then apposed the character to a bright-yellow background to create contrast. This visual was adapted with 3 other figures (a middle-aged man, a teenage boy, and a teenage girl) to illustrate that people could suffer from post–COVID-19 regardless of their age or gender.

We used these visuals on billboards, flyers, posters, and slides to communicate and advertise the RAFAEL platform and chatbot technology on all the communication channels that were made available via the Geneva University Hospitals. These channels included the hospital’s website (intranet and for external communication), social networks (Facebook, Twitter, Instagram, LinkedIn, and YouTube), and ads or digital posters at the hospital and at medical and digital health conferences.

The communication campaign was continuous (online posting via the Geneva University Hospitals). Newsletters, flyers, and posters were used to announce each monthly webinar and adapted to the theme of the webinar. These were distributed and used by the Association of Medical Physicians in Geneva, the University of Geneva, the Swiss Medical Review, and the specific platforms and outreach of each RAFAEL partner for communication. The Geneva University Hospitals additionally used paid campaigns on Facebook and Instagram to increase the scope of communication.

### Data Collection

Data were collected during the use of the chatbot (Table 4). A survey will also be launched after the deployment phase, via a link that will redirect participants to a secure Research Electronic Data Capture (REDCap, Vanderbilt University) questionnaire collecting information, if they wish to participate.

**Table 4:** Outcomes measures.

Outcome measure		Method
**Use of the RAFAEL chatbot**		
	Number of unique users	Chatbot platform
	Number of new users	Chatbot platform
	User profile	Chatbot platform
	Interactions (buttons, open messages)	Chatbot platform
	Interactions per user	Chatbot platform
	Webchat displays	Webpage traffic, chatbot platform
	Volume of intents	Chatbot platform
	Volume of stories	Chatbot platform
	Matching rate	Chatbot platform
	Fallback rate	Chatbot platform
	Use of contact form	Chatbot platform
	Overall satisfaction level	Chatbot platform
**Themes and requests**		
	Type of information requested (adult, pediatric)	Chatbot platform
	Themes (general information, medical information, social impact, symptom specific)	Chatbot platform
	Frequency of requests per theme	Chatbot platform
	Matching rate per theme	Chatbot platform
	Satisfaction level per theme	Chatbot platform
**Survey**		
	Sociodemographics (age, sex, profession, location)	Survey
	Overall rating of the RAFAEL chatbot (score=0-10)	Survey
	Qualitative assessment	Survey (see Multimedia Appendix 1)
	Quantitative assessment	Survey (see Multimedia Appendix 1)

### Data Analysis

Data are automatically analyzed by the chatbot (number of users, interactions, matching). A descriptive analysis of user participation and the collected data will be computed using percentages and frequencies for continuous and categorical variables. The chi-square or Fischer exact test (where appropriate) results will be analyzed by the frequency of themes, the profile of users by theme, and the satisfaction rate by theme. The survey results will be used to analyze usage, themes, and satisfaction by age, sex, profession, geographic location, and education level. Data analysis will also consider the use over time and satisfaction and match rates over time.

A qualitative thematic analysis will be conducted for the open-ended questions in the user satisfaction survey to evaluate the most common themes and answers of users with regard to satisfaction, information needs, information acquisition, accuracy, and ease of use of the RAFAEL chatbot.

## Results

### Summary

Development of the digital platform started in 2021. Beta testing with focus groups was conducted in October 2021 for the adult section, and deployment was in November 2021. Beta testing with focus groups for the pediatric section was conducted in January 2022.

The adult beta testing groups included 10 participants, and the results showed a matching rate of 71.7% (43/60) between the intents and answers and a 73.3% (44/60) positive user feedback rate. The pediatric beta testing groups included 5 adolescent participants and 5 adult participants (parents). The results showed a 76.7% (46/60) matching rate and a 71.7% (43/60) positive user feedback rate.

Use of the RAFAEL chatbot and platform was driven by the webinars as well as communication campaigns, with an uptake in users in the 48 hours following each monthly webinar as well as following the communication campaigns. Results of the communication campaigns showed an interest by the media and other academic and governmental institutions that contacted us in order to learn more about the chatbot technology and join the RAFAEL platform.

Between November 2021 and January 2023, the chatbot had 30,488 interactions and 5807 unique users, with 5.1 interactions per user, on average. Details of the stories, matching rate, and overall satisfaction level are presented in Table 5 and of themes and requests in Table 6.

**Table 5:** Results of the RAFAEL chatbot from November 2021 to January 2023 (unique users, N=5807; interactions, N=30,488; stories, N=8061).

Use of the RAFAEL chatbot		Users, n (%)
**Unique adult users**		
	I think I have post–COVID-19.	2589 (44.6)
	I have been diagnosed with post–COVID-19.	1107 (19.1)
	Other.	498 (8.6)
	I am a health care professional.	297 (5.1)
	Profile not selected.	861 (14.8)
**Unique pediatric users**		
	I think my child has post–COVID-19.	195 (3.4)
	My child has been diagnosed with post–COVID-19.	91 (1.6)
	Other.	43 (0.7)
	I am a health care professional.	42 (0.7)
	Profile not selected.	84 (1.4)
**Interactions**		
	Buttons	22,876 (75.0)
	Open-ended messages	7612 (25.0)
	Interactions per user	5.1
Chatbot matching rate		6417 (79.6)
Fallback		1507 (18.7)
Use of the contact form		356 (4.4)
Overall satisfaction (positive feedback) level (feedback provided, n=2451 users)		1795 (73.2)

**Table 6:** Results of the RAFAEL chatbot regarding themes and requests (N=8061) from November 2021 to January 2023.

Themes and requests		Users, n (%)
**Symptoms**		5612 (69.6)
	Fatigue	1255 (22.4)
	Loss or change of smell	554 (9.9)
	Headache	539 (9.6)
	Pain	389 (6.9)
	Dyspnea	382 (6.8)
	Chest pain	350 (6.2)
	Difficulty concentrating	291 (5.2)
	Gastrointestinal symptoms	221 (3.9)
	Dizziness	239 (4.3)
	Sleeping disorders	197 (3.5)
	Cough	200 (3.6)
	Tinnitus	137 (2.4)
	Psychiatric symptoms	146 (2.6)
	Paresthesias	145 (2.6)
	Dermatologic symptoms	128 (2.3)
	Fever	106 (1.9)
	Loss or change of voice	105 (1.9)
	Palpitations	103 (1.8)
	Visual disorders	72 (1.3)
	Dysautonomic symptoms	39 (0.7)
General information		510 (6.3)
Screening		174 (2.2)
Diagnosis		168 (2.1)
Consultation		598 (6.4)
**Treatment**		527 (6.5)
	General treatment	388 (73.6)
	Pharmacologic treatment	47 (8.9)
	Neuro-reeducation	30 (5.7)
	Physical therapy	36 (6.8)
	Complementary medicine	26 (4.9)
Vaccination		153 (1.9)
Social impact and social support		84 (1.0)
Pathophysiology and research		34 (0.4)
Other		201 (2.5)

## Discussion

### Principal Findings

The RAFAEL chatbot and platform are an innovative and accurate information dissemination and learning tool addressing population concerns on post–COVID-19 in children and adults. The RAFAEL chatbot considers medical safety in applying this technology and makes users aware that the tool is not a medical consultation, while providing them with accurate and up-to-date information. Community engagement, communication, and partnerships were essential tools in driving traffic and use. Preliminary results showed that users in Geneva and the French-speaking world were interested in this type of technology to address their concerns and seek answers. Users were also satisfied with the answers given and the technology used, with high matching and satisfaction rates.

The RAFAEL chatbot provides reliable and accurate information. The fact that the RAFAEL chatbot builds on the ongoing experience of the CoviCare research group [2,9,11], the post–COVID-19 pediatric and adult consultations [14,15] at the Geneva University Hospitals, and the partnerships with patients, the community, and partner institutions has been key in predefining triggers, algorithms, and adequate responses. Its use has been promoted by the need in the population, the ease of accessibility via smartphones and computers, the ergonomic design, and the continuous monitoring of information in the initial phases to correct answers rapidly, when needed, and answer users via the contact form in the case of fallback. Its use has also been promoted by the accompanying communication campaigns, as information dissemination is not possible without an invitation and initial communication to use the technology provided. Communication remains 1 of the essential tools in establishing the success of any new technology and enables users and the bidirectional exchange to continue improving and feeding the database.

The use of machine learning could enable us to quickly and accurately deploy scalable, low-cost, and efficient tools in health care [31]. This could be particularly useful when trying to learn about a specific or new subject or disease [32]. In the context of post–COVID-19, this was a tool that was “just in time” [33], with several of the elements surrounding the disease and its impact still to be defined. When citizens and health care professionals faced the challenge of a lack of full understanding of a medical condition, chatbot technology helped deploy and disseminate all the information available in an accurate and easily accessible language, while underlining the learning approach of the chatbot and the fact that users were helping improve the knowledge base by providing information in a participative patient- and citizen-based approach. The chatbot technology was particularly useful in a time of limited resources, where health care professionals could not absorb the increasing demand during and between pandemic waves. This underlines the importance of developing and integrating helpful tools in the clinical care pathway so that patients have access to information, increased awareness, potential self-management strategies, and a decreased need for in-person consultation or in-person contact. Physicians and health care professionals should, however, be mindful of a potential digital divide, with the risk of inaccessibility for elderly patients or individuals without internet access or with difficulty in adopting these tools. The use of this large, scalable approach potentially reaching most of the population could help dedicate the more traditional resources (consultations, phone resources) to those without access to chatbot technology or with complex cases necessitating a more human or personalized approach. This could even eventually facilitate access to information for all groups as each approach will be tailored to specific needs and used in the correct and most efficient way.

### Limitations

To date, the RAFAEL chatbot is still in its learning phase, and further evaluation is needed to assess the advantages and disadvantages of this technology and apply it to other fields in medicine and chronic conditions. As the RAFAEL chatbot acts as a medical and information chatbot, its limitations lie in the anonymity and degree of privacy provided, also limiting its anthropomorphic capabilities when compared to a human being. To stay as anonymous and deidentifying as possible, the RAFAEL chatbot cannot be a substitute for a medical teleconsultation. Chatbots are, however, an asset when their use is well defined in the clinical care pathway, thus reducing the need for medical consultations and dedicating those to patients who require more specific information or management. The RAFAEL chatbot was designed in Switzerland and deployed worldwide in the francophone world, with its first version available in French. Eventually, 1 of the limitations of the RAFAEL chatbot will be its use in different languages and different cultural and societal contexts. The partnerships put in place help the RAFAEL platform overcome these challenges, and these elements are being considered when developing and expanding the RAFAEL chatbot.

### Comparison With Other Work

Chatbots have been used in the acute COVID-19 response for risk assessment, surveillance, information dissemination, eligibility screening, and vaccination strategies [16]. Chatbots have also been used, even if not frequently to date, for chronic diseases [20]. The response in emergency and acute settings is usually different from the response in the case of chronic diseases, considering the patient population, the timeline, and the approach to management. Post–COVID-19 requires both an orientation and a coordination approach (large-scale, quick response) as well as a long-term approach addressing the social and medical impacts of a chronic condition on individuals and the community. Using the RAFAEL chatbot to address these challenges is a new and innovative way to be readily available with accurate information based on the evidence to date, with limited resources. With its self-learning ability, low cost, and minimally required maintenance and monitoring, chatbot technology could rely on a project management team (developers, communication experts, medical and social experts) and its expanding partnerships to answer a large numbers of users, therefore reducing in-person contact and reserving it for complex and specific situations.

### Conclusion

The RAFAEL chatbot is, to the best of our knowledge, the first chatbot developed to address post–COVID-19, a chronic condition affecting millions of individuals. The evolution, underlying mechanisms, and management options of post–COVID-19 are still being clarified. The use of a scalable tool for information dissemination and the use of a machine learning system to acquire and better define population concerns represent an innovative approach to address these concerns. Lessons learned from the RAFAEL chatbot could further encourage a participative approach to learning, help develop this technology, and apply it to other chronic conditions, medical needs, and public health needs in times of limited resources.

## Appendix

Multimedia Appendix 1 Satisfaction survey for chatbot users.

## Abbreviations

ICRC: International Committee of the Red Cross
NLP: natural language processing
UNHCR: United Nations High Commissioner for Refugees
