# Supplementary material for: The Development of a Chatbot Technology to Disseminate Post–COVID-19 Information: Descriptive Implementation Study
Source: J Med Internet Res. 2023 Jun 5;25:e43113. doi: 10.2196/43113 (PMC10246662; doi:10.2196/43113)
Supplement: Multimedia Appendix 1 [file jmir_v25i1e43113_app1.docx]

**Appendix 1. Satisfaction Survey for Chatbot users**

| **Question** | **Answers** | **Sub-question** |
| --- | --- | --- |
| **Sociodemographic information** |  |  |
| Age |  |  |
| How do you mostly identify? | Male  Female  Non-binary  Prefer not to answer | |
| Education level | Primary  Secondary  Tertiary  Prefer not to answer | |
| Employment status | Salaried  Retired  Independent worker  Unemployed  Homemaker  Disability  Student  Prefer not to answer | |
| Occupation | Architecture and engineering  Arts, culture and entertainment  Business, management and administration  Communications  Community and social services  Education  Science and technology  Installation, repair and maintenance  Farming, fishing and forestry  Government  Health and medicine  Law and public policy  Sales  Prefer not to answer | |
| How would you rate your Chatbot experience? | Score 0-10 | |
| What are you hoping to learn from the Chatbot? | Open-ended | |
| Has the Chatbot been able to answer your questions or request? | Yes  No  Somewhat | Add open-ended explanation |
| What other information do you wish to receive from the Chatbot? | Open-ended |  |
| Where would you have searched for this information if the Chatbot was not available? | Online websites  Online social network groups  Online: other sources -> specify  My doctor  Public health helpline -> specify  Medical helpline -> specify  My friends or family  I would not have searched for it  Other -> specify | |
| By using the Chatbot, were you able to avoid using another source of information that you would have used otherwise? | Yes  No  Somewhat | |
| Have you used a Chatbot before? | Yes  No  Cannot remember | If yes, how does this experience compare to previous ones? |
| How do you rate the response time of the Chatbot? | Excellent  Good  Neither good nor bad  Unsatisfactory  Very unsatisfactory | Add open-ended detail explanation |
| Do you have any suggestions? | Open-ended | |
